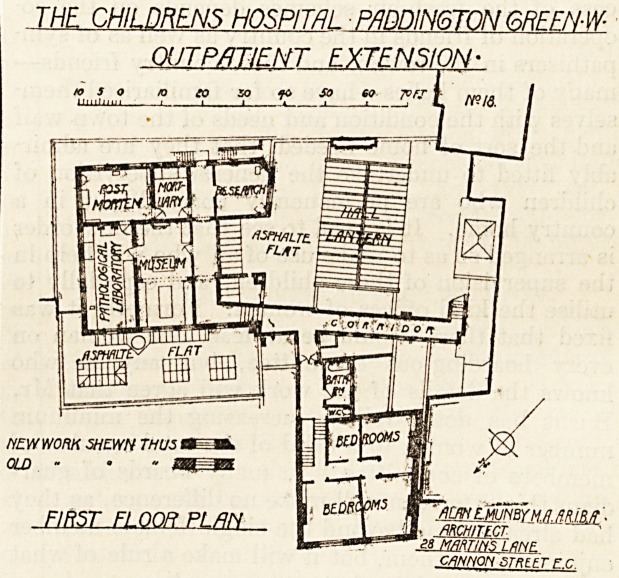# Children's Hospital, Paddington Green

**Published:** 1911-11-18

**Authors:** 


### HOSPITAL ARCHITECTURE AND CONSTRUCTION.

[Communications on this subject should be marked "Architecture" in the left-hand top corner of the envelope.]

### Children's Hospital, Paddington Green.

### EXTENSION OF OUT-PATIENT DEPARTMENT.

The extensions shown on the plan consist of the enlargement and improvement of the out-patient department, the provision of an entirely new pathological department, additional bedrooms for nurses, and some improvements in the lighting of the basement and communication between the front and the back of the buildings. It is a little difficult to follow on the plan what the extent of the out-patient department was before the alterations were made; but it is evident that the conditions under which the work had to be done must have been very restricted indeed. The alterations have been ingeniously contrived, and the architect has made the most of the space at his disposal; but the shape of the site is a peculiarly difficult one, and it was hardly to be expected that absolute perfection should be achieved.

### The Main Entrance.

The entrance for patients is by a passage eight feet wide in Church Street; some little distance in on the right is a covered space for perambulators. Passing through a lobby with which the receiving room for casualties and the observation-room both communicate, the patients enter the main waiting hall. Immediately to the left of the entrance is a recess where the sister in charge or casualty officer sits and divides the patients into their respective classes as medical or surgical cases. The waiting hall is roughly a parallelogram, with an area of some 2,000 feet. On one side is a wide open space, around either side of which the consulting-rooms are grouped. These comprise two rooms for the physicians, with a dressing-room common to both; and one room for the surgeon, with anæsthetic, operation, and recovery rooms all *en suite*. Next to the recovery room is the observation room, with the exit door to the entrance lobby referred to above. This is not quite an ideal position for this room and it is difficult to see how it can be adequately lighted, and it ought to be provided with an exit directly into the open air. It is clear, however, that this could not be arranged here, and the success or failure of the plan will depend entirely on careful administration. The receiving-room, which is intended for casualties, has direct communication with the operation-room and the recovery-room. Patients on leaving the consulting-rooms either cross the hall to the almoner's room or go direct to the passage leading to the medicine waiting-room, and so by a separate exit to the street. This arrangement involves a certain amount of cross-traffic, but here again the difficulties of the site prevent any other arrangement.

### The Pathological Department.

The pathological department is on the first floor over the consulting-rooms, and consists of a mortuary, postmortem-room, pathological laboratory, museum, and research laboratory. The means of access to this department is apparently by a corridor over the waiting hall conected with the main block, and this corridor has direct communication with the new bedrooms for nurses. On the face of it this does not seem a very desirable arrangement, but as it appears that the new nurses' rooms are really part of an old house in Church Street adapted for the purpose, the arrangement must be regarded as a temporary one only.

The architect by whom these alterations were planned is Mr. Alan E. Munby, A.R.I.B.A.

## Figures and Tables

**Figure f1:**
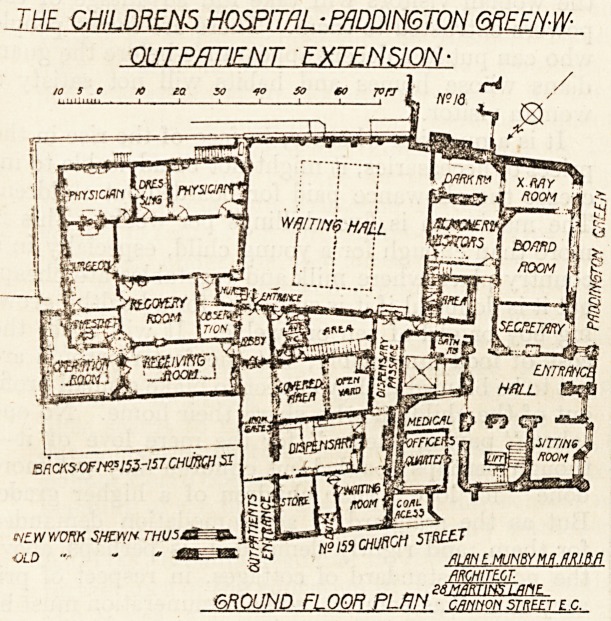


**Figure f2:**